# Personalization of Aspirin Therapy Ex Vivo in Patients with Atherosclerosis Using Light Transmission Aggregometry

**DOI:** 10.3390/diagnostics10110871

**Published:** 2020-10-26

**Authors:** Hamzah Khan, Reid C. Gallant, Abdelrahman Zamzam, Shubha Jain, Sherri Afxentiou, Muzammil Syed, Zachary Kroezen, Meera Shanmuganathan, Philip Britz-McKibbin, Margaret L. Rand, Heyu Ni, Mohammed Al-Omran, Mohammad Qadura

**Affiliations:** 1 Division of Vascular Surgery, St. Michael’s Hospital, Toronto, ON M4B 1B3, Canada; hamzah.khan@mail.utoronto.ca (H.K.); abdelrahman.zamzam@unityhealth.to (A.Z.); jains@ucalgary.ca (S.J.); Sherri.Afxentiou@unityhealth.to (S.A.); muzammil.syed@mail.utoronto.ca (M.S.); mohammed.al-omran@unityhealth.to (M.A.-O.); 2 Keenan Research Centre for Biomedical Science, Li Ka Shing Knowledge Institute of St. Michael’s Hospital, Toronto, ON M4B 1B3, Canada; reid.gallant@mail.utoronto.ca (R.C.G.); Heyu.Ni@unityhealth.to (H.N.); 3 Department of Chemistry and Chemical Biology, McMaster University, Hamilton, ON L8S 4M1, Canada; kroezezp@mcmaster.ca (Z.K.); shanmm2@mcmaster.ca (M.S.); britz@mcmaster.ca (P.B.-M.); 4 Department of Laboratory Medicine & Pathobiology, University of Toronto, Toronto, ON M4B 1B3, Canada; margaret.rand@sickkids.ca; 5 Departments of Biochemistry and Pediatrics, University of Toronto, Toronto, ON M4B 1B3, Canada; 6 Translational Medicine, Research Institute, Division of Haematology/Oncology, The Hospital for Sick Children, Toronto, ON M4B 1B3, Canada; 7 Department of Surgery, University of Toronto, Toronto, ON M4B 1B3, Canada

**Keywords:** aspirin, light transmission aggregometry, non-sensitivity, personalized medicine, atherosclerosis, anti-platelet, resistance

## Abstract

Acetylsalicylic acid (ASA), also known as aspirin, appears to be ineffective in inhibiting platelet aggregation in 20–30% of patients. Light transmission aggregometry (LTA) is a gold standard platelet function assay. In this pilot study, we used LTA to personalize ASA therapy ex vivo in atherosclerotic patients. Patients were recruited who were on 81 mg ASA, presenting to ambulatory clinics at St. Michael’s Hospital (*n* = 64), with evidence of atherosclerotic disease defined as clinical symptoms and diagnostic findings indicative of symptomatic peripheral arterial disease (PAD), with an ankle brachial index (ABI) of <0.9 (*n* = 52) or had diagnostic features of asymptomatic carotid arterial stenosis (CAS), with >50% stenosis of internal carotid artery on duplex ultrasound (*n* = 12). ASA compliance was assessed via multisegmented injection-capillary electrophoresis-mass spectrometry based on measuring the predominant urinary ASA metabolite, salicyluric acid. LTA with arachidonic acid was used to test for ASA sensitivity. Escalating ASA dosages of 162 mg and 325 mg were investigated ex vivo for ASA dose personalization. Of the 64 atherosclerotic patients recruited, 8 patients (13%) were non-compliant with ASA. Of ASA compliant patients (*n* = 56), 9 patients (14%) were non-sensitive to their 81 mg ASA dosage. Personalizing ASA therapy in 81 mg ASA non-sensitive patients with escalating dosages of ASA demonstrated that 6 patients became sensitive to a dosage equivalent to 162 mg ASA and 3 patients became sensitive to a dosage equivalent to 325 mg ASA. We were able to personalize ASA dosage ex vivo in all ASA non-sensitive patients with escalating dosages of ASA within 1 h of testing.

## 1. Introduction

Effective platelet inhibition in atherosclerotic patients is critical to prevent the progression of atherosclerosis and adverse cardiovascular events [1,2]. Antiplatelet therapy, specifically acetylsalicylic acid (ASA) or aspirin, is considered first line therapy for the prevention of cardiovascular events in several atherosclerotic vascular diseases, including symptomatic peripheral arterial disease (PAD) and carotid artery stenosis (CAS) [3,4,5,6]. For instance, the Antithrombotic Trialists Collaboration demonstrated a 22% reduction in mortality and serious adverse vascular events in patients taking ASA [7].

Current clinical guidelines suggest prescribing 81–325 mg of ASA for the management of symptomatic PAD and CAS patients [3,4,5]. However, it has been documented that approximately 30% of patients suffer from “aspirin non-sensitivity”—a phenomenon where ASA fails to inhibit platelet aggregation. Some studies even suggest aspirin non-sensitivity is prevalent in up to 60% of the population [8,9,10,11]. Furthermore, it has been demonstrated that ASA non-sensitive patients are at a higher risk of adverse cardiovascular events, with 39% of ASA non-sensitive patients suffering from a cardiovascular events compared to 16% of ASA sensitive patients [12].

Currently, the exact mechanism behind ASA non-sensitivity is not completely understood; however, it can be described as either pharmacokinetic or pharmacodynamic in nature [13,14]. Pharmacokinetic non-sensitivity is caused by insufficient concentrations of ASA reaching patient platelets in order to completely inhibit platelet aggregation. This could be due to malabsorption, increased rate of ASA metabolism and excretion, or increased rate of platelet turnover, among others [13,14]. Pharmacodynamic non-sensitivity will occur when enough ASA reaches the patient platelets; however, due to genetic polymorphisms in the cyclooxygenase (COX-1) enzyme, ASA is unable to effectively acetylate the serine residue in its active site and inhibit platelet aggregation [13,14].

Previous research has demonstrated that many patients will not have an antiplatelet response to 81 mg ASA [8,9,10,11,12,15,16], with poor compliance often attributed to half of all cases of apparent ASA non-sensitivity [17]. It is also evident that personalization of anti-platelet therapy in cardiac patients undergoing percutaneous coronary intervention can lead to the reduction in net clinical adverse events; however, data in the PAD and CAS population is lacking [18,19,20,21,22]. Therefore, a need exists for an algorithm for a personalized medical approach that will ensure optimal dosing for patients [18]. In this pilot study, we established an ex vivo method using light transmission aggregometry (LTA) with the platelet agonist arachidonic acid to personalize ASA dosage in patients who are non-sensitive to 81 mg ASA, while also confirming compliance to recent aspirin intake based on urinary salicyluric acid (SU) screening.

## 2. Experimental Section

### 2.1. Ethics Approval

This study was performed in accordance with the Declaration of Helsinki and approved by the Unity Health Toronto Research Ethics Board at St Michael’s Hospital in Toronto, Toronto, ON, Canada (REB #16-375, 8 February 2017). Informed consent was obtained from all participants.

### 2.2. Patient Selection

In this pilot study, consecutive patients with documented symptomatic PAD and asymptomatic CAS on 81 mg ASA, attending the Vascular Surgery Outpatient Clinic at St Michael’s Hospital between November 2018 and November 2019, were invited to participate. Patients with PAD were defined as patients with an ankle brachial index (ABI) <0.9 or toe-brachial index (TBI) <0.67 and abnormal distal pulses with claudication. Patients with asymptomatic CAS were defined to have >50% stenosis of the internal carotid artery on duplex ultrasound in the absence of neurological symptoms. The following patients were excluded: (1) recent diagnosis (within 12 months) of acute coronary syndrome (ACS), cerebral vascular attack (CVA), or transient ischemic attack (TIA); (2) need for: dual antiplatelet therapy, antiplatelet therapy other than ASA, or oral anticoagulant therapy; (3) history of drug or alcohol abuse; (4) history/active diagnosis of thrombocytopenia, bleeding, or coagulopathy; (5) patients presenting with acute limb ischemia; (6) pregnant/breastfeeding women; (7) patients unable to provide written informed consent. Patients not taking 81 mg ASA, and patients without evidence of atherosclerotic disease taking 81 mg ASA were also recruited as controls (see Appendix A).

### 2.3. Baseline Measurements

Patients were questioned about their medical history and underwent a complete physical examination. Type II Diabetes Mellitus (DM) was defined as glycosylated hemoglobin A1c ≥ 6.5% or the use of anti-diabetic medication. Hyperlipidemia was defined as total cholesterol >5.2 mmol/L or triglyceride >1.7 mmol/L or the use of anti-hyperlipidemic medication. Hypertension was defined as systolic blood pressure ≥130 mmHg or diastolic pressure ≥80 mmHg or the use of antihypertensive medication [23]. Renal disease was defined as an estimated glomerular filtration rate less than 60 mL/min/1.73 m^2^ as per the Kidney Disease Outcomes Quality Initiative 2002 guidelines. Smoking status was recorded for each patient.

### 2.4. Blood Sample Collection and Light Transmission Aggregometry

**Figure 1:**
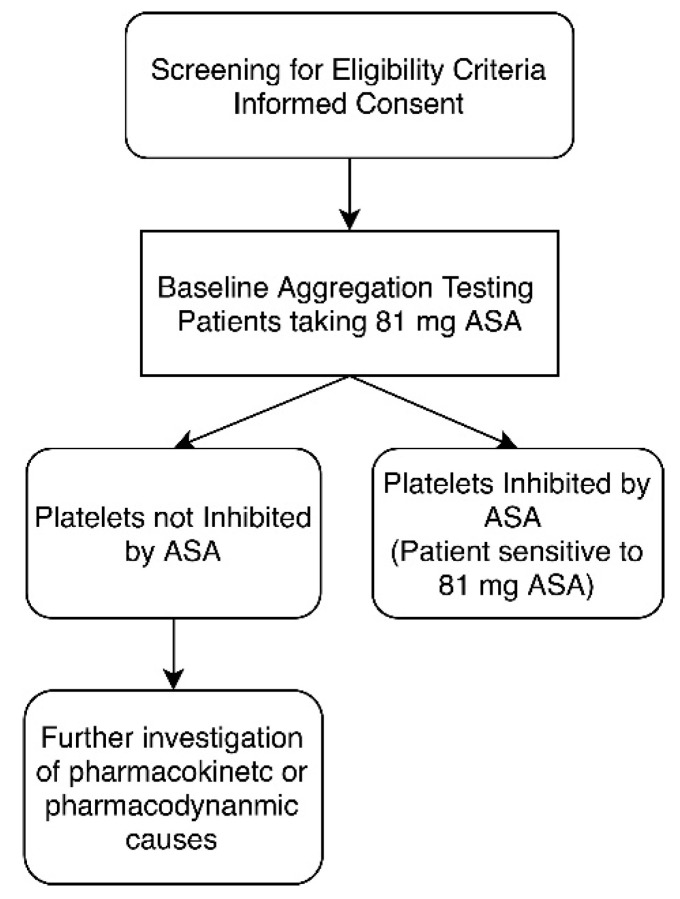
Study design for aspirin (acetylsalicylic acid (ASA)) sensitivity testing. Once patient eligibility was established, each patient underwent light transmission aggregometry (LTA) testing to establish ASA sensitivity. Platelets were considered not inhibited by ASA if there was evidence of ≥20% maximal platelet aggregation after activation with arachidonic acid.

Blood samples were drawn into vacutainer tubes containing sodium citrate. Each sample was prepared as previously described [24]. In summary, platelet-rich plasma (PRP) was prepared by centrifugation of whole blood at 300× *g* for 7 min and platelet-poor plasma (PPP) was obtained by additional centrifugation for 10 min at 1500× *g*. Maximal platelet aggregation (light transmission) of 100% was defined as transmission through PPP and 0% defined as light transmission through PRP. Platelet concentrations within PRP were adjusted to 2–3 × 10^6^/mL using autologous PPP [24,25]. Platelets were activated using 0.5 mg/mL lyophilized arachidonic acid reconstituted in distilled water (101297, Bio/Data Corporation, Horsham, PA, USA) at 37 °C, with a stir rate of 1000 rpm, and measured for 10 min. Platelet aggregation was monitored by a computerized Chrono-log aggregometer (Chrono-Log Corporation, Havertown, PA, USA) [26,27]. Patients were considered to have platelets not inhibited by 81 mg ASA if they had a ≥20% maximal platelet aggregation after activation with arachidonic acid, as described in previous literature (Figure 1) [25,28,29,30,31,32,33]. As a control, to ensure the viability of platelets, additional samples were activated with 5 µM adenosine diphosphate (ADP) reconstituted in distilled water [24,34,35].

### 2.5. Urine Salicyluric Acid Analysis

Salicyluric acid (SU) concentrations, the major urinary drug metabolite of ASA, were determined to assess compliance with ASA therapy. Urine samples were collected from each patient at their baseline visit. Samples were aliquoted and stored at −80 °C prior to analysis. Urine samples were thawed slowly on ice and then diluted 5-fold in deionized water containing two internal standards (200 µM 2-naphthelenesulfonate, 860 µM creatinine-d3). All reagents and chemical standards were purchased from Sigma–Aldrich Inc. (Oakville, ON, Canada), and all analyses were performed using an Agilent G7100A capillary electrophoresis (CE) instrument coupled to an Agilent 6230 time-of-flight mass spectrometer (TOF-MS) system with a dual coaxial sheath liquid Jetstream electrospray source (Agilent Technologies Inc., Santa Clara, CA, USA). Multisegment injection-capillary electrophoresis-mass spectrometry (MSI-CE-MS), using a 13 sample plug serial sample injection configuration [36], was used to simultaneously quantify urinary concentrations of creatinine and SU. The CE instrument was operated under normal polarity with an applied voltage of 30 kV at 25 °C. The TOF-MS was set for the negative ion detection mode, where data was acquired at a rate of 500 ms/spectrum with full scan data acquisition from *m*/*z* 50–1700. The TOF-MS fragmentor, skimmer, and Oct1 RF were set to 120, 65, and 750 V, respectively. The electrospray ionization conditions were Vcap = 2000 V, nozzle voltage = 2000 V, nebulizer gas = 10 psi, drying gas = 8 L/min at 300 °C, and sheath gas flow = 3.5 L/min at 195 °C. External calibration curves for creatinine (0.5–5.0 mM) and SU (5–200 µM) were measured in triplicate at six concentrations with good linearity (*R*^2^ > 0.994) and precision (CV < 8%), and their ion responses were normalized to creatinine-d3 and 2-naphthelenesulfonate, respectively. Urinary metabolites were identified based on their accurate mass and co-migration after spiking urine samples with authentic standards by MSI-CE-MS. SU concentrations (µM) were also normalized to creatinine (mM) in order to adjust for differences in hydration status when relying on single-spot urine samples [37]. Patients with SU concentrations >27 µM (or >5.25 µg/mL) were used as a minimum cut-off indicative of drug compliance, reflecting recent intake of ASA due to potential non-ASA sources of salicylates from dietary exposures [38,39].

### 2.6. Ex Vivo Aspirin Personalization

**Figure 2:**
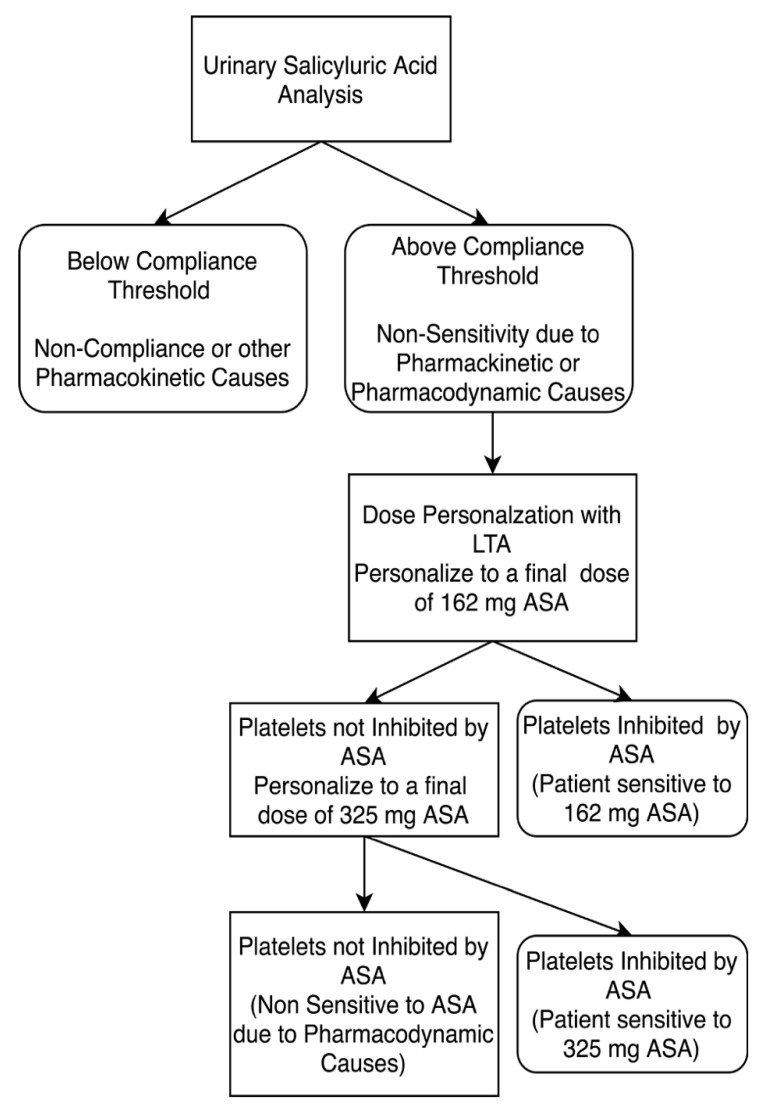
Study design for personalizing aspirin therapy in 81mg ASA compliant patients. Patients who were non-sensitive to 81 mg ASA therapy underwent ASA dose personalization at final doses equivalent to 162 and 325 mg ASA. Platelets were considered not inhibited by ASA if there was evidence of ≥20% maximal platelet aggregation during LTA analysis after activation with arachidonic acid. ASA compliance was defined as urinary salicyluric acid levels >27 µM.

ASA dosage personalization was carried out on patients compliant with ASA therapy who retained platelet aggregation despite 81 mg ASA during their baseline measurement (Figure 1 and Figure 2). Dosage personalization was achieved by spiking fresh PRP samples with 10 or 30 µM ASA to obtain a final ASA equivalent dosage of 162 mg or 325 mg ASA, respectively. The ex vivo concentrations are approximately equivalent to those seen in plasma after ingestion of respective dosages [13,40,41]. Each spiked sample was incubated at 37 °C for 15 min before testing the platelet aggregation response to arachidonic acid via LTA, as described above.

### 2.7. Statistical Analysis

Demographics and clinical characteristics were summarized and reported for the study population. Continuous variables were tested for normality using the Shapiro–Wilk test and normality plots. Normally distributed continuous variables were summarized and reported in terms of mean and standard deviation. For non-normally distributed data, the median and interquartile ranges (IQRs) were calculated. Categorical variables were reported as counts and percentages. Percentages were calculated according to the number of patients for whom data were available. Independent *t*-tests or the Mann–Whitney U test was used to calculate the significance between continuous variables, and Fisher’s exact test or a chi-square test was used for categorical variables. All analyses were carried out at a 5% two-sided significance level. GraphPad Prism software, version 8.4.2, was used for data analysis.

## 3. Results

### 3.1. Cohort Description

A total of 64 atherosclerotic patients on 81 mg ASA met the study criteria and were enrolled in this study. Of the 64 recruited patients, 52 patients had documented PAD, while the other 12 patients had clinical and diagnostic features of asymptomatic CAS. The study cohort was comprised of 54% males, and a high prevalence of cardiovascular risk factors (hypertension, hypercholesterolemia, diabetes, smoking, and history of coronary arterial disease) was observed (Table 1).

### 3.2. Baseline ASA Sensitivity Testing

**Figure 3:**
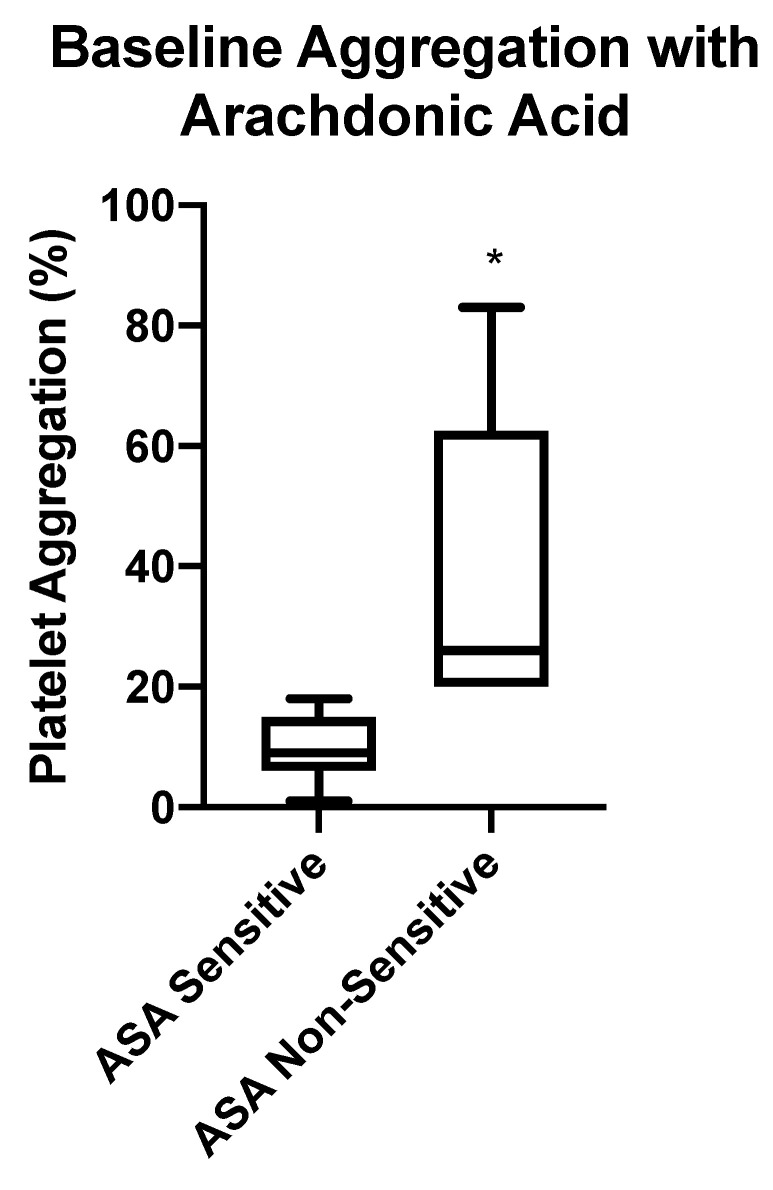
Baseline aspirin (ASA) sensitivity testing using LTA. Box-whisker plots depicting maximum platelet aggregation in response to arachidonic acid (AA) in patients taking 81 mg ASA. Patients were considered ASA non-sensitive if there was ≥20% maximal platelet aggregation. Significant difference of maximum aggregation between ASA sensitive and non-sensitive patients is represented by (*) with *p* < 0.0001.

Of the 64 atherosclerotic patients recruited to the study, 47 patients (73%) were sensitive to their 81 mg ASA therapy. The remaining 17 patients (27%) had ≥20% maximal platelet aggregation in response to arachidonic acid, indicating that platelets were not inhibited by ASA and may be non-sensitive to 81 mg ASA (Figure 3).

### 3.3. Investigating Possible Mechanisms for Lack of Platelet Inhibition by ASA

**Figure 4:**
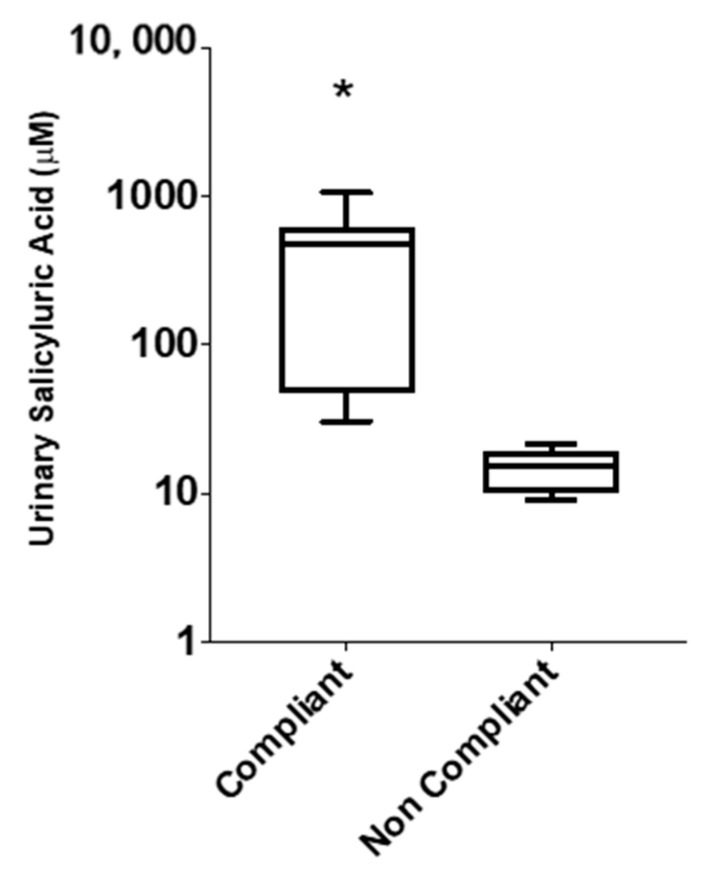
Box-whisker plots depicting distribution of urinary salicyluric acid (SU) concentrations in ASA compliant (≥27 µM, *n* = 9) and ASA non-compliant (<27 µM, *n* = 8) patients, based on absolute concentrations (µM) measured from single-spot urine samples. Significant difference of SU levels between compliant and non-compliant patients is represented by (*) with *p* < 0.05.

In order to investigate some of the reasons behind the lack of platelet inhibition in those 17 patients who were taking 81 mg ASA, we assessed for ASA compliance. This was achieved by measuring SU levels in patients’ urine samples based on a recommended minimum cut-off threshold of 27 µM SU [17], which accounts for dietary intake of salicylates [39]. Figure 4 shows a box-whisker plot for measured urinary SU concentrations (µM) from patients who had platelets not inhibited by ASA (*n* = 17). Our data shows that 8 of the 17 patients were ASA non-compliant, defined as <27 µM, while the remaining 9 patients were considered ASA compliant, defined as >27 µM. As expected, there was significantly higher, yet variable, urinary SU levels measured among ASA compliant patients, with a median concentration of 473 µM compared to 15 µM in non-compliant patients. This biological variance reflects differences in urine hydration status, ASA pharmacokinetics, and sampling times when relying on single-spot urine specimens. SU concentrations were also normalized to creatinine (µmol/mmol), which generated similar outcomes in terms of classifying ASA compliant and ASA non-compliant patients.

**Figure 5:**
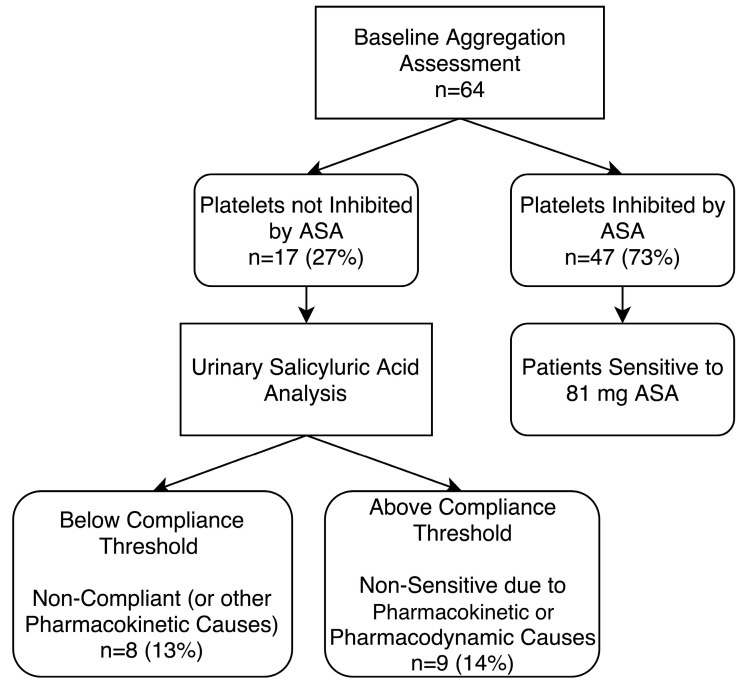
Baseline aggregation testing of patients on 81 mg aspirin (ASA) and urinary salicyluric acid assessment to determine non-compliance or non-sensitivity due to pharmacokinetic or pharmacodynamic causes. Platelets were considered not inhibited by ASA if there was evidence of ≥20% maximal platelet aggregation during light transmission aggregometry (LTA) after activation with arachidonic acid. ASA compliance was confirmed by urinary salicyluric acid concentrations >27 µM.

Therefore, for all recruited patients (*n* = 64), 13% (8 of 64) were determined to be non-compliant to prescribed ASA at the time of sampling, while 14% (9 of 64) were determined to be compliant but non-sensitive to 81 mg ASA due to pharmacokinetic and/or pharmacodynamics causes (Figure 5).

### 3.4. ASA Dosage Personalization

**Figure 6:**
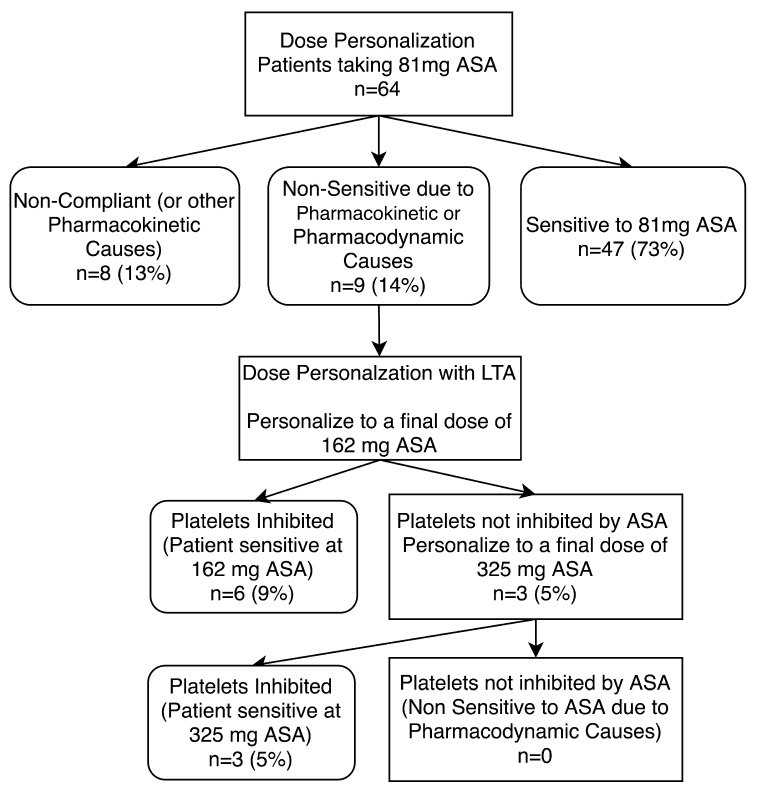
Dose personalization of 81 mg aspirin (ASA) compliant patients. Patients non-sensitive to 81 mg ASA therapy underwent ASA dose personalization at 162 and 325 mg doses. Platelets were considered not inhibited by ASA if there was evidence of ≥20% maximal platelet aggregation during LTA analysis after activation with arachidonic acid. ASA compliance was defined as urinary salicyluric acid levels >5.25 µg/mL. Percentages shown are in comparison to all patients recruited to the study (*n* = 64).

**Figure 7:**
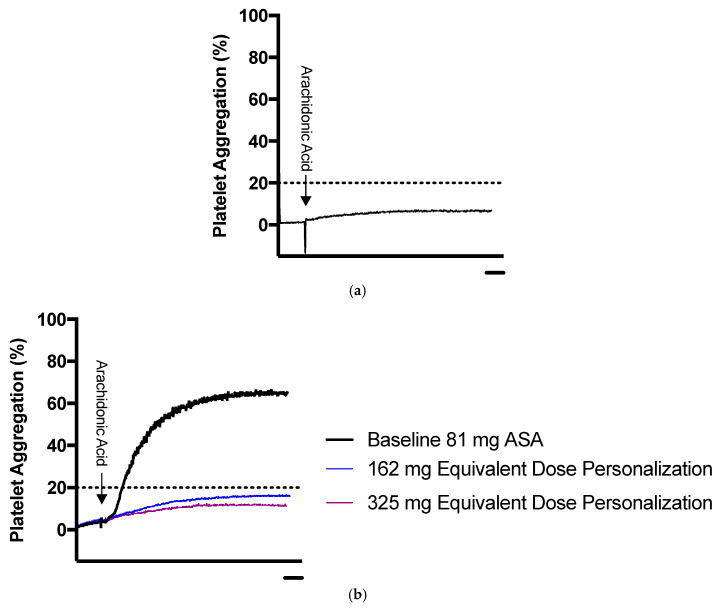
Sample representation of platelet aggregation results for ASA dose personalization using LTA. (**a**) Representative platelet aggregation curve of a patient sensitive to 81 mg ASA. (**b**) Representative platelet aggregation curve for patient non-sensitive to 81 mg ASA undergoing dose personalization. The blue line represents maximum platelet aggregation in platelet rich plasma spiked ex vivo with 162 mg equivalent ASA, and the purple line represents platelet rich plasma spiked ex vivo with 325 mg equivalent ASA. The dotted line represents the ASA sensitivity cut-off. Scale bars: 1 minute.

In an attempt to personalize ASA dosage in the 9 ASA compliant but non-sensitive patients, we spiked fresh PRP samples from the patients with 10 µM ASA, producing a final equivalent dose of 162 mg ASA. This was followed by LTA analysis. We observed that 6 patients (9%) became sensitive to 162 mg ASA, while 3 patients (5%) remained non-sensitive to 162 mg ASA. In a final attempt to overcome ASA non-sensitivity in the remaining 3 patients, a third fresh PRP sample was spiked with 30 µM of ASA, producing a final equivalent dose of 325 mg ASA. Once again, this was followed by LTA analysis, as previously described. Our final LTA analysis demonstrated that these 3 patients became sensitive at 325 mg ASA (Figure 6). Thus, all 9 non-sensitive patients started responding to ASA with increased dosages (Figure 7).

## 4. Discussion

In this pilot study, we found that 27% of patients had platelets that were not inhibited by 81 mg ASA. Of these patients, almost half of them were not compliant with their 81 mg ASA therapy at the time of sampling, while the remaining half were compliant but non-sensitive. Our data demonstrated that ASA non-sensitivity was overcome ex vivo via ASA personalization in all patients. We have outlined an algorithm that can be used for personalizing a patient’s ASA dosage within 1 h.

Identifying and treating the underlying cause(s) of aspirin non-sensitivity is a challenging task, as there are many contributing factors to ASA non-sensitivity. Some of these include non-compliance, under-dosage, malabsorption, adverse drug interactions, increased platelet turnover, and genetic polymorphisms of the COX-1 enzyme. However, several studies have demonstrated that aspirin non-sensitivity can be overcome [29,42,43,44]. For instance, an analysis of the ASPECT study showed that higher ASA dosage (up to 325 mg) was effective in reducing aspirin non-sensitivity in diabetic patients with coronary arterial disease [45]. Similarly, other studies demonstrated clinical benefits at higher ASA doses (up to 325 mg) compared to doses below 75 mg, but doses exceeding 500 mg were associated with a greater incidence of gastrointestinal side effects, such as bleeding, without any additional clinical benefit [46,47,48]. However, one limitation of these previous studies is that they tested a higher ASA dose on all patients, rather than just those who were non-sensitive to the low dose. This meant that 70% of patients received a higher dosage of ASA when not required. In our study, we demonstrated that all patients became sensitive at doses up to 325 mg. Interestingly, no patients were non-sensitive at 325 mg, demonstrating that no patients had COX-1 polymorphisms that lead to pharmacodynamic insensitivity in our population. We demonstrated the efficacy of a simple algorithm that can assist physicians in identifying and possibly overcoming ASA non-sensitivity in patients facing this issue.

Previous research has demonstrated a wide range of values for ASA non-sensitivity in the population [46,47,48]. However, a major limitation found in the majority of these studies is the lack of an established method for ensuring compliance with therapy. Patients may have been non-compliant to their ASA but refrained from disclosing it to their physicians/researchers. In this study, MSI-CE-MS was used to objectively measure the major urinary ASA metabolite, SU, to ensure that patients were compliant, reflecting recent intake of ASA [39,49]. Our data indicated that 13% of recruited patients on ASA were non-compliant, representing a significant fraction of patients not taking their prescribed ASA. Compliance to ASA therapy is vital to prevent adverse cardiovascular events, and our data suggests that physicians need to better communicate the importance of compliance to medical therapy to their patients. However, it should be noted that these non-compliant patients, as per our urine analysis, may have been compliant but, due to other pharmacodynamic causes, such as malabsorption of ASA, lead to a lack of higher SU concentrations within urine. Due to the complexity of ASA compliance testing, a reliable compliance monitoring method has been lacking in most previous ASA non-sensitivity research, typically consisting of pill counts or self-reports by patients. These are often not standardized methods, and patients still may be non-compliant but may hide pills or falsely disclose to their physician their regular compliance. This may be one of the several reasons why some research has demonstrated ASA non-sensitivity as high as 60% in patients. Here, we were able to objectively measure the SU levels within patient’s urine to confirm that ASA metabolites were present and ensure ingestion of ASA. 

We have shown that personalizing ASA therapy is effective for overcoming 81 mg ASA non-sensitivity ex vivo. Within a relatively short period of time, we were able to determine the dosage of ASA that would best benefit the patient. Using this simple approach, physicians have the potential to identify their ASA non-sensitive patients and subsequently personalize and determine an appropriate dosage for their patient. This methodology may also reduce the risk of gastric bleeding at higher ASA dosage, as patients who are sensitive at 81 mg will not be prescribed a higher dose. A study by Yeomans et al. demonstrated that increasing low dose ASA from 75–100 mg to 101–325 mg is associated with a three-fold increase in the incidence of gastric ulcers. Therefore, it is necessary to only increase the dose if it is clinically evident that a patient is non-sensitive to 81mg ASA [50].

Several studies have attempted to elucidate the risk factors associated with ASA non-sensitivity. Cohen et al. demonstrated that diabetes and obesity had a significant association. However, the sample size used by the authors was fairly small [51]. Similarly, Shen et al. conducted a large study in 745 patients and demonstrated a significant association between ASA non-sensitivity and being older, female, having significantly higher total cholesterol and low-density lipoprotein C (LDL-C) levels, lower hematocrit, and higher platelet counts. However, this study was conducted with only relatively healthy patients [52]. Gum et al. [29] also observed a similar trend of higher rates of ASA non-sensitivity in older ages and females. Interestingly, they also reported that smokers were less likely to be ASA non-sensitive, in stark contrast to Cao et al. [53], who noted the opposite trend. A meta-analysis by Krasopoulous et al. [12], of 20 studies, analyzed 2930 patients and concluded that only females and those with renal impairment were more likely to be ASA non-sensitive. They showed no association between age, hypertension, diabetes, dyslipidemia, smoking, or other comorbidities with ASA non-sensitivity. A study by Lee et al. [54] similarly noted that being female and having renal insufficiency are risk factors for ASA non-sensitivity. Finding a clear picture of the risk factors significantly associated with ASA non-sensitivity is still required, as they are often inconsistent between studies and methods of ASA non-sensitivity testing.

The COMPASS trial demonstrated that patients taking combination therapy of 2.5 mg rivaroxaban twice daily, in addition to low dose 81 mg ASA, had significantly lower rates of adverse cardiovascular events when compared with patients on low dose ASA alone or full dose rivaroxaban [55]. However, patients on combination therapy had a significantly higher risk of major bleeding [55]. Future trials may look into personalizing therapy in ASA non-sensitive patients by adding 2.5 mg rivaroxaban twice daily in combination with low dose ASA and assessing any change in clinical events and ASA sensitivity.

Our pilot study has some limitations. First, we included only a modest number of patients, which limited power to adjust for confounders. Out of the 64 recruited patients, 9 patients were personalized ex vivo. Further studies with a larger sample size will be beneficial to further determine the efficacy of this algorithm of ASA dose personalization. Second, this is an ex vivo study and thus is difficult to predict whether a patient would have a similar response in vivo. Routine antiplatelet testing is also not recommended by clinical guidelines and is often limited to special cases. However, with this pilot study, we hope to establish an algorithm that can be used in future studies to establish a recommended routine antiplatelet test for ASA sensitivity. Lastly, some patients may have difficulty in absorbing ASA in the gut, which could lead to reduced ASA levels in the blood and a false assumption that these patients are non-compliant.

This pilot study establishes a foundation for future directions, including a clinical trial to personalize patient ASA doses in vivo. A longitudinal study design would also elucidate whether personalization prevents adverse cardiovascular events while also minimizing gastric toxicity of ASA.

## 5. Conclusions

It is important for physicians to monitor both ASA sensitivity and compliance. ASA non-sensitivity is a clinically relevant issue, effecting a large population of patients worldwide. With a comprehensive test for identifying ASA non-sensitivity and personalizing therapy, it would be possible to reduce surgical intervention failure and adverse cardiovascular events, such as heart attacks, strokes, and death. Not only can the methodology presented here be used to personalize therapy ex vivo in patients taking 81 mg ASA, it can also be used to optimize an ASA dose in individual patients who have not yet initiated their ASA regime. This method of personalizing ASA doses will prevent increased risk of bleeding with higher doses of ASA, as only non-sensitive patients will be prescribed a higher ASA dose. This methodology was successful in reversing aspirin non-sensitivity and could be used by physicians to prescribe personalized ASA doses in hospitals within one hour of blood sample collection. However, larger clinical trials are required to demonstrate if this method of personalization translates to in vivo ASA response.

## Figures and Tables

**Table 1:** Baseline characteristics of subjects on 81 mg ASA with documented atherosclerotic disease.

Characteristics	Atherosclerotic Patients on 81 mg ASA (*n* = 64)
Mean (SD)	
ABI	0.6 ± 0.19
Age (yrs)	71 ± 8
Platelet Count (10^3^/μL)	252 ± 62
Frequency (%)	
Sex (% Male)	54
Hypertension (%)	71
Hyperlipidemia (%)	88
Diabetes (%)	45
Renal Insufficiency (%)	7
Smokers (%)	88
CAD (%)	32
Patients taking a Statin (%)	73
Patients taking an ACEi/ARB (%)	50
Patients taking a BB (%)	27
Patients taking a CCB (%)	20

Ankle brachial index (ABI); acetylsalicylic acid (ASA); coronary artery disease (CAD); angiotensin-converting enzyme (ACE) inhibitors (ACEi/Arb); beta blockers (BB); calcium channel blockers (CCB). Continuous variables are shown as mean ± standard deviation; all numbers were rounded to one decimal place. Categorical variables are shown as percentages; all numbers were rounded up with zero decimal places.

**Figure A1:**
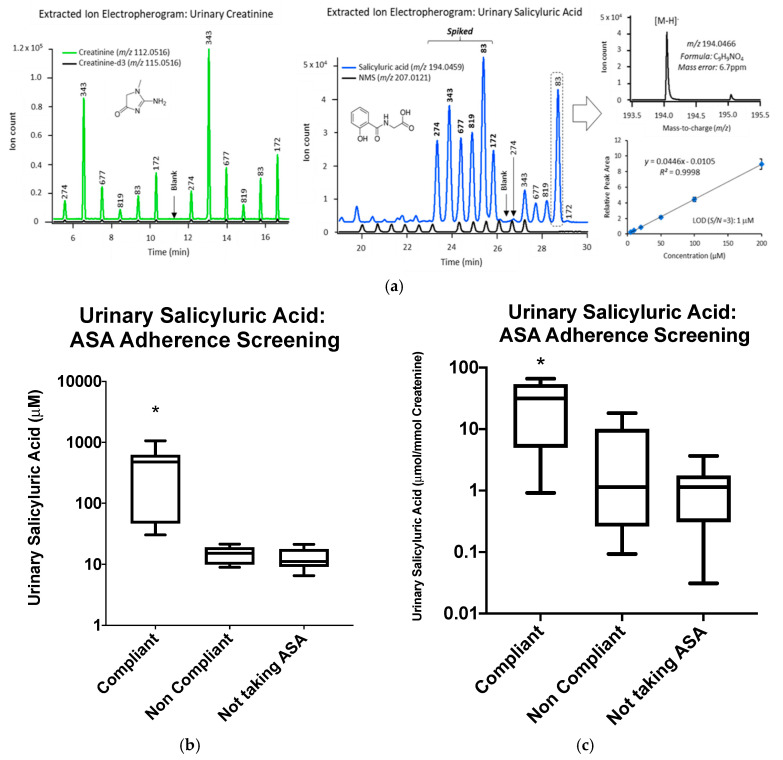
(**a**) Extracted ion electropherograms for multiplexed analyses of salicyluric acid (SU) and creatinine simultaneously by multisegmented injection-capillary electrophoresis-mass spectrometry (MSI-CE-MS) under negative ion mode conditions, which were confirmed by their accurate mass and co-migration after spiking urine samples with authentic standards. (**b**) SU analysis of 17 patients with platelets not inhibited by 81 mg ASA and 12 controls not taking 81 mg ASA. (**c**) Concentration distributions for SU normalized to creatinine (µmol/mmol) in 17 patients with platelets not inhibited by 81 mg ASA and 12 controls not taking 81 mg ASA. Significant difference of SU levels between compliant and non-compliant patients is represented by (*) with *p* < 0.05.

## Appendix A

### Appendix A.1. Methodology

As a positive control, patients with no evidence of atherosclerotic disease who had been taking 81 mg ASA were recruited. As a negative control, patients with documented PAD or CAS (as described above) who had not taken ASA, or any other antiplatelet within the past 2 weeks, were also recruited. The exclusion criteria are as described previously in Section 2.

### Appendix A.2. Results

A total of 26 additional patients were recruited to the study, consisting of 12 atherosclerotic patients not taking ASA or any other antiplatelet therapy and 14 non-atherosclerotic patients taking 81 mg ASA (Table A1).

**Table A1:** Baseline characteristics of patients not taking 81 mg ASA with documented atherosclerotic disease and patients taking 81 mg ASA with no documented atherosclerotic disease.

Characteristics	Atherosclerotic Patients on Not Taking ASA (*n* = 12)	Non-Atherosclerotic Patients on 81 mg ASA (*n* = 14)
Mean (SD)		
ABI	0.63 ± 1.9	1.07 ± 0.2
Age (yrs)	73 ± 11	65 ± 17
Platelet Count (10^3^/μL)	304 ± 79	231 ± 56
Frequency (%)		
Sex (% Male)	83	86
Hypertension (%)	83	43
Hyperlipidemia (%)	83	57
Diabetes (%)	25	14
Renal Insufficiency (%)	8	7
Smokers (%)	83	71
CAD (%)	8	0
Patients taking a Statin (%)	67	50
Patients taking an ACEi/ARB (%)	58	28
Patients taking a BB (%)	8	29
Patients taking a CCB (%)	17	14

Ankle brachial index (ABI); acetylsalicylic acid (ASA); coronary artery disease (CAD); angiotensin-converting enzyme (ACE) inhibitors (ACEi/Arb), beta blockers (BB); calcium channel blockers (CCB). Continuous variables are shown as mean ± standard deviation; all numbers were rounded to one decimal place. Categorical variables are shown as percentages; all numbers were rounded up with zero decimal places.

Of the 14 non-atherosclerotic patients recruited to the study, 13 patients were sensitive to their 81 mg ASA therapy. The remaining 1 person (7%) had ≥20% maximum platelet aggregation in response to arachidonic acid, indicating that their platelets were not inhibited by ASA. After SU analysis, it was determined that the patient was non-compliant, with SU levels below the compliance threshold of 27 µM urinary SU. As expected, all 12 atherosclerotic patients not taking ASA had active platelets in response to arachidonic acid.

### Appendix A.3. Urinary Salicyluric Acid Analysis

As controls, urine samples from the 12 patients not taking 81 mg ASA were analyzed alongside the 17 patients with platelets not inhibited by ASA, as described above. As expected, all patients not taking 81 mg ASA were below the threshold levels of urinary SU. There was also no statistically significant difference between ASA non-compliant patients and control patients not taking 81 mg ASA (Figure A1b). Results were normalized to creatinine in order to adjust for differences in hydration status when relying on single-spot urine samples (Figure A1c).
